# Identification of differentially expressed genes and signaling pathways in Gaoyou duck ovary at different physiological stages

**DOI:** 10.3389/fvets.2023.1190998

**Published:** 2023-05-03

**Authors:** Lei Zhang, Jun Xie, Guobo Sun, Rongchao Ji, Xiaoming Li, Xue Zhang, Jian Wang

**Affiliations:** School of Animal Science, Jiangsu Agri-animal Husbandry Vocational College, Taizhou, China

**Keywords:** Gaoyou duck, transcriptome, ovarian development, egg production, differentially expressed genes

## Abstract

**Introduction:**

Gaoyou duck is famous in China and abroad for its good production of double-yolk eggs. However, there has been no systematic research on the egg-laying characteristics of the Gaoyou duck, which limits the development and utilization of breed resource.

**Methods:**

To identify the essential genes related to ovarian development, the transcriptome profiles of the ovaries of Gaoyou ducks at different physiological stages were analyzed. The transcriptome profiles of the ovaries of Gaoyou ducks at 150 d (before laying), 240 d (egg laying) and 500 d (nesting) were constructed, and the differentially expressed genes (DEGs) underwent GO (gene ontology) and KEGG (Kyoto Encyclopedia of Genes and Genomes) analyses.

**Results:**

The 6 randomly selected DEGs were verified by real-time fluorescent quantitative PCR that their relative expression was consistent with the transcriptional expression profile. Furthermore, KEGG analysis found that 8 candidate signaling pathways were essential for ovarian development, including the MAPK signaling pathway, Progesterone-mediated oocyte maturation, Cell adhesion molecules (CAMs), NOD-like receptor signaling pathway, ECM-receptor interaction, Focal adhesion, TGF-beta signaling path-way and Phagosome. Finally, 5 key DEGs were identified to participate in ovarian development, including TGIF1, TGFBR2, RAF1, PTK2 and FGF10.

**Discussion:**

Our findings reveal the mechanisms under-lying the molecular regulation of related genes in Gaoyou duck ovarian development.

## 1. Introduction

The egg-laying performance of poultry is reportedly closely related to ovarian tissue. The ovary is an important reproductive organ of poultry in which folliculogenesis, selection, maturation, and ovulation occur and represents the endocrine gland that synthesizes and secretes estrogen (1). Its function directly affects the level of egg-laying performance of poultry. Therefore, poultry breeders usually select the ovary to study the egg-laying traits and identify differences related to these traits from the histological and molecular levels to explore the regulatory mechanism of the egg-laying performance (2, 3). In recent years, the ovarian tissues of high- and low-egg-production individuals of poultries, including ducks, geese, and chicken, have been studied through RNA-Seq, which provides a convenient way to explore the candidate genes related to egg-laying performance. Karippadakam et al. (4) compared the high and low egg-laying ovarian transcriptome of Indian domestic ducks and found 38 DEGs potentially related to egg production performance. Kun et al. (5) conducted a comparative study on high and low egg-laying Leizhou black ducks using ovary tissue, revealing two key signaling pathways, steroid biosynthesis and FSH signal pathway, involved in the egg-laying performance of Leizhou black ducks. Sun et al. (6) conducted a comparative study on high and low egg-laying Shan-ma ducks using ovarian tissues, revealing ITGB2, ITGB5, and ITGA8 are candidate genes for the egg-laying performance of Shan-ma duck.

**Figure 1:**
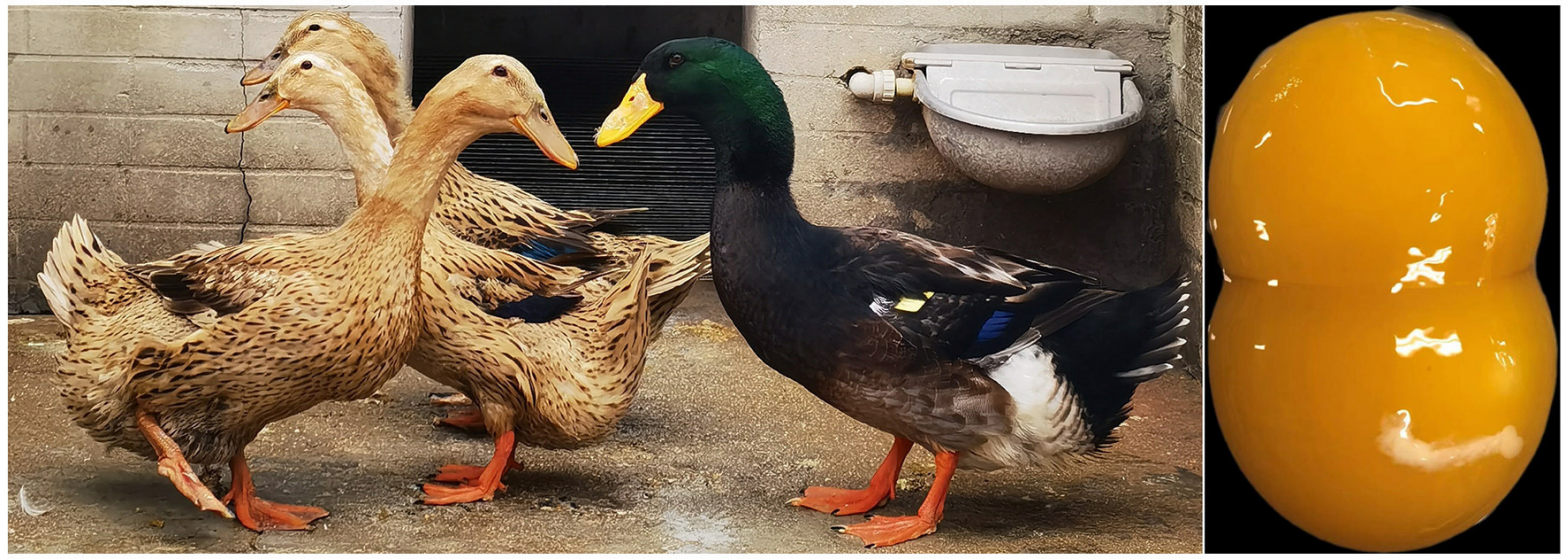
The phenotypic characteristics of Gaoyou duck and its double-yolk egg.

Gaoyou duck, also known as the Gaoyou shelduck, ranks first among the three famous ducks in China. Its origin is Gaoyou City, Jiangsu Province, China (Figure 1). It has been raised for more than 100 years in the Yangtze-Huaihe region of China. It is an excellent local poultry variety for meat and egg production (7, 8). Gaoyou duck has the advantages of roughage resistance, strong foraging ability, suitable for grazing and breeding, fast growth and development, good egg quality, etc. It is famous in China and abroad for its good production of double-yolk eggs, estimated to be up to 3.5% (9). However, until now, there has been no systematic observation and research on the egg-laying characteristics of the Gaoyou duck, which limits the development and utilization of this breed resource.

This study performed a comparative transcriptomic analysis of the Gaoyou duck ovary at different physiological stages (before egg laying, egg laying, and nesting) to identify the differentially expressed genes and signaling pathways involved in regulating ovarian function and follicular development of the Gaoyou duck.

## 2. Materials and methods

### 2.1. Animals and sampling

This study was approved by the Institutional Animal Ethics Committee of Jiangsu Agri-animal Husbandry Vocational College, Taizhou, Jiangsu, China. A total of 260 female Gaoyou ducks were raised under the same recommended environmental and nutritional conditions at the National Gene Bank of Waterfowl Resources (Jiangsu, China). At 150 days (before laying, BL), 240 days (egg laying, EL), and 500 days (nesting, NE), three ducks were randomly selected for each day. Ducks were, then, sacrificed at different stages through exsanguination. Samples from the ovarian tissue were collected after removing the egg yolk. All samples above were snap-frozen in liquid nitrogen for follow-up experiments. All animal procedures were performed according to the guidelines provided by the China Council on Animal Care, and the protocols were approved by the Experimental Animal Ethics Committee of Jiangsu Agri-animal Husbandry Vocational College (Ethic approval file No. JAHV-2020-58).

### 2.2. RNA isolation and sequencing

Total RNA was extracted from ovarian tissue using a total RNA Kit (Ambion, Austin, TX, United States) in accordance with the manufacturer's instructions. RNA integrity and concentration were evaluated using NanoDrop (NanoDrop, Thermo Scientific) and Agilent 2100 Bio-analyzer (Agilent Technologies, Palo Alto, CA, United States). All the qualified RNA samples were then transported to OE Biotech. Co. (Shanghai, China) for transcriptome sequencing. The Illumina HiSeq 2500 system sequencing platform was used.

### 2.3. Quantitative reverse transcription polymerase chain reaction (qRT-PCR) analysis

To validate the RNA-Seq results, the expression of six randomly selected DEGs was assessed using qRT-PCR. Total RNA was extracted from ovary samples using TRIzol Kit (Invitrogen). The primers (Supplementary Table 1) were designed using Primer 5.0 software. qPCR was performed on an ABI 7500 Real-time Detection System (Applied Biosystems). The relative gene expression levels of selected DEGs were quantified based on GADPH gene expression by the 2^−ΔΔCt^ method.

### 2.4. Transcriptome data analysis

Raw reads of each sample were obtained from Illumina sequencing, and clean reads were obtained by Trimmomatic software (10) to make the whole quality control process of each sample. Then, the clean reads were mapped to the duck reference genome (BGI duck 1.0 reference) for the downstream analysis as previously described (6). Genes with an absolute log_2_|fold-change|≥1.50 and adjusted *P* ≤ 0.05 were differentially expressed genes (DEGs) in this study. The gene ontology (GO) database was, then, used to identify the functions of the DEGs (5, 11). Kyoto Encyclopedia of Genes and Genomes (KEGG) pathways were assigned to classify unigenes using the online KEGG Automatic Annotation Server (http://www.genome.jp/kegg/kaas/) (12). The sequencing raw data have been deposited into the Sequence Read Archive in NCBI with accession number PRJNA940302.

### 2.5. Statistical analysis

All statistical analyses in this study were performed using SPSS version 19.0 (IBM, Armonk, NY, United States). The results were expressed as mean ± standard deviation. Differences were assessed using the independent sample *t*-test. A *P* < 0.05 was statistically significant.

## 3. Results

### 3.1. Sequencing data statistics

A total of nine ovarian tissue transcriptome libraries at different physiological stages (before egg laying, egg laying, and nesting) were constructed. The results are shown in Table 1 and Supplementary Table 2. Both the raw reads and clean reads of each library were more than 49 million. To ensure the quality of the data, quality control and filtering were performed on the data, and high-quality clean reads were obtained by removing the adaptor reads and low-quality reads. The Q30 was >92%, and the GC content was >47%. The clean reads were mapped to the reference genome (*Anas platyrhynchos*) with a total map ranging from 75.93% to 82.38%. The above sequencing data analysis showed that the data quality was reliable and could be used for further analyses.

**Table 1:** Preprocessed results of samples' sequencing data.

**Samples**	**Total reads**	**Total mapped reads**	**Mapped ratio**	**Uniquely mapped reads**	**Uniquely mapped ratio**
BL1	547,123,32	480,381,54	87.80%	448,564,85	81.99%
BL2	543,039,64	476,136,14	87.68%	446,097,11	82.15%
BL3	544,073,86	479,042,14	88.05%	448,207,67	82.38%
EL1	506,125,22	421,564,36	83.29%	384,277,84	75.93%
EL2	504,618,32	419,718,02	83.18%	384,509,23	76.20%
EL3	495,311,74	418,797,34	84.55%	383,233,44	77.37%
NE1	563,145,48	485,779,62	86.26%	451,347,59	80.15%
NE1	578,846,80	511,490,05	88.36%	480,049,99	82.93%
NE1	525,245,50	454,180,67	86.47%	424,387,86	80.80%

### 3.2. Analysis of differentially expressed genes (DEGs)

**Figure 2:**
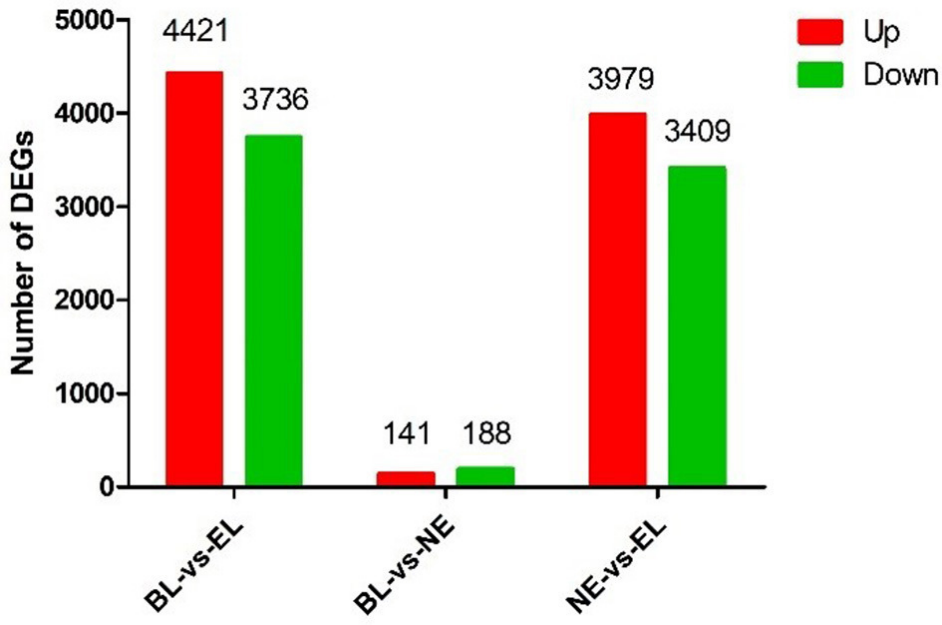
Differentially expressed genes in the ovary of Gaoyou duck at different physiological stages.

**Figure 3:**
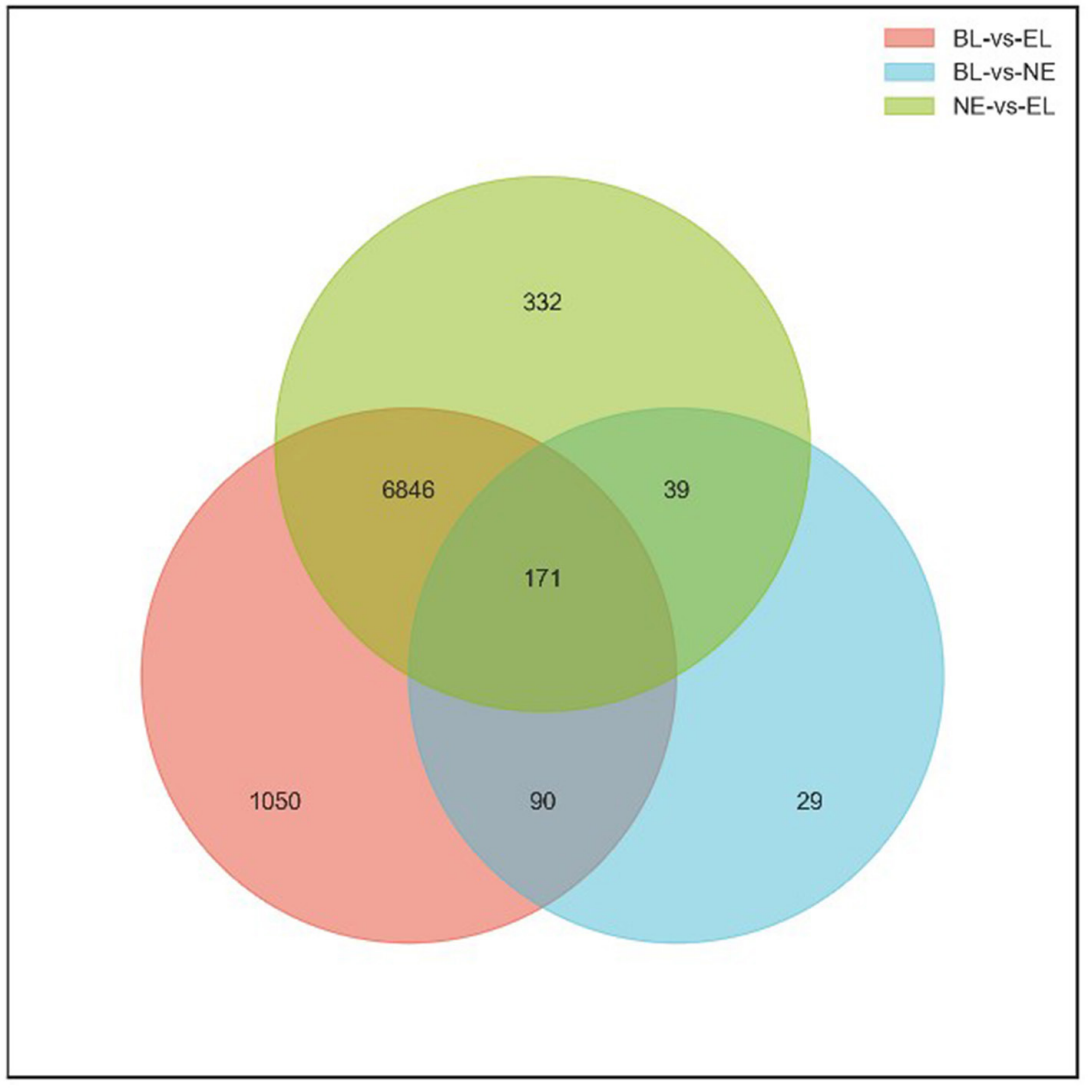
Venn diagram of DEGs showing overlap among the three comparisons.

Based on the nine ovarian tissue samples at three different physiological stages used for the sequencing, statistical analysis of the expression results was conducted by pairwise comparisons. As shown in Figure 2, a total of 8,157 genes (4,421 upregulated and 3,736 downregulated) were differentially expressed between BL and EL, with only 329 DEGs (141 upregulated and 188 downregulated) differentially expressed between BL and EL, and 7,388 DEGs (3,979 upregulated and 3,409 downregulated) expressed between NE and EL. A comparative analysis of DEGs obtained at three different physiological stages of Gaoyou duck showed that the intersection of DEGS during the pairwise comparisons of BL-vs.-EL, BL-vs.-NE, and NE-vs.-EL yielded 171 co-expressed DEGs (Figure 3), with 1,050, 29, and 332 DEGs expressed in BL-vs.-EL, BL-vs.-NE, and NE-vs.-EL, respectively. To focus on the candidate DEGs for ovarian follicular development of Gaoyou duck at different physiological stages, the top 10 DEGs for each pairwise comparison were screened for further analysis, among which one DEG represented both in BL-vs.-EL and BL-vs.-NE, and data are shown in Table 2.

**Table 2:** Top 10 DEGs in each comparison group.

**BL-vs-EL**		**BL-vs-NE**		**NE-vs-EL**	
**Gene ID**	**Log**_2_ **FC**	**Gene ID**	**Log**_2_ **FC**	**Gene ID**	**Log**_2_ **FC**
LOC113842444	−11.65	LOC113840293	−6.51	LOC113844140	−10.80
LOC101797788	−11.03	GPNMB	−6.26	LOC113841006	−9.92
SMAGP	−9.95	TYR	−6.10	GH1	−8.06
NEU4	−9.62	LOC106020550	−3.84	PRL	−7.90
LOC101799867^1^	−9.29	IL22RA2	−3.62	LOC101799867^1^	−7.62
ZBBX	16.68	LOC113844165	2.57	LOC110352021	12.39
LOC106014422	16.92	LOC106015682	2.58	LOC101798434	13.44
GUCA1B	17.03	LOC101805447	2.64	LOC101798128	14.58
LOC106015402	17.69	PROK2	2.74	LOC113839763	16.08
SYPL1	18.27	LOC101802349	3.18	SFTPB	16.24

^1^represents repeat DEG both in BL-vs.-EL and NE-vs.-EL; log_2_FC represent log_2_ fold change.

### 3.3. Validation of RNA-Seq results using qRT-PCR

**Figure 4:**
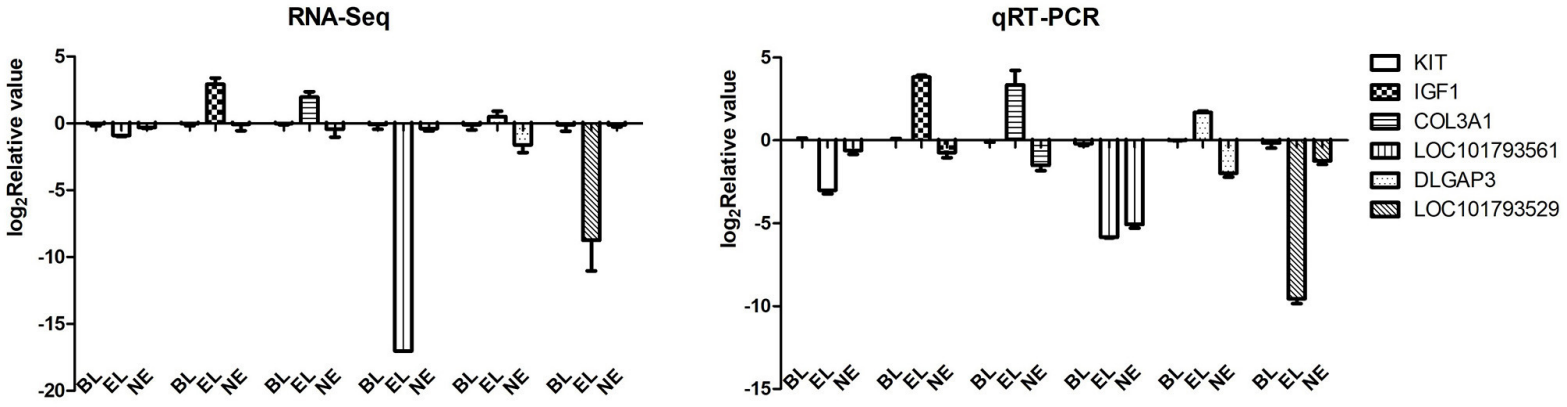
RNA-Seq validation using qRT-PCR. A total of six DEGs were selected randomly to test the accuracy of RNA sequencing.

To verify the expression levels of DEGs observed in our RNA-seq analysis, six randomly selected DEGs from transcriptomic sequencing were validated by qPCR. The qPCR results showed a similar trend in the expression of these genes, indicating that transcriptomic sequencing data and candidate DEGs from RNA-Seq had high reliability and accuracy (Figure 4).

### 3.5. Gene Ontology (GO) and KEGG analyses

**Figure 5:**
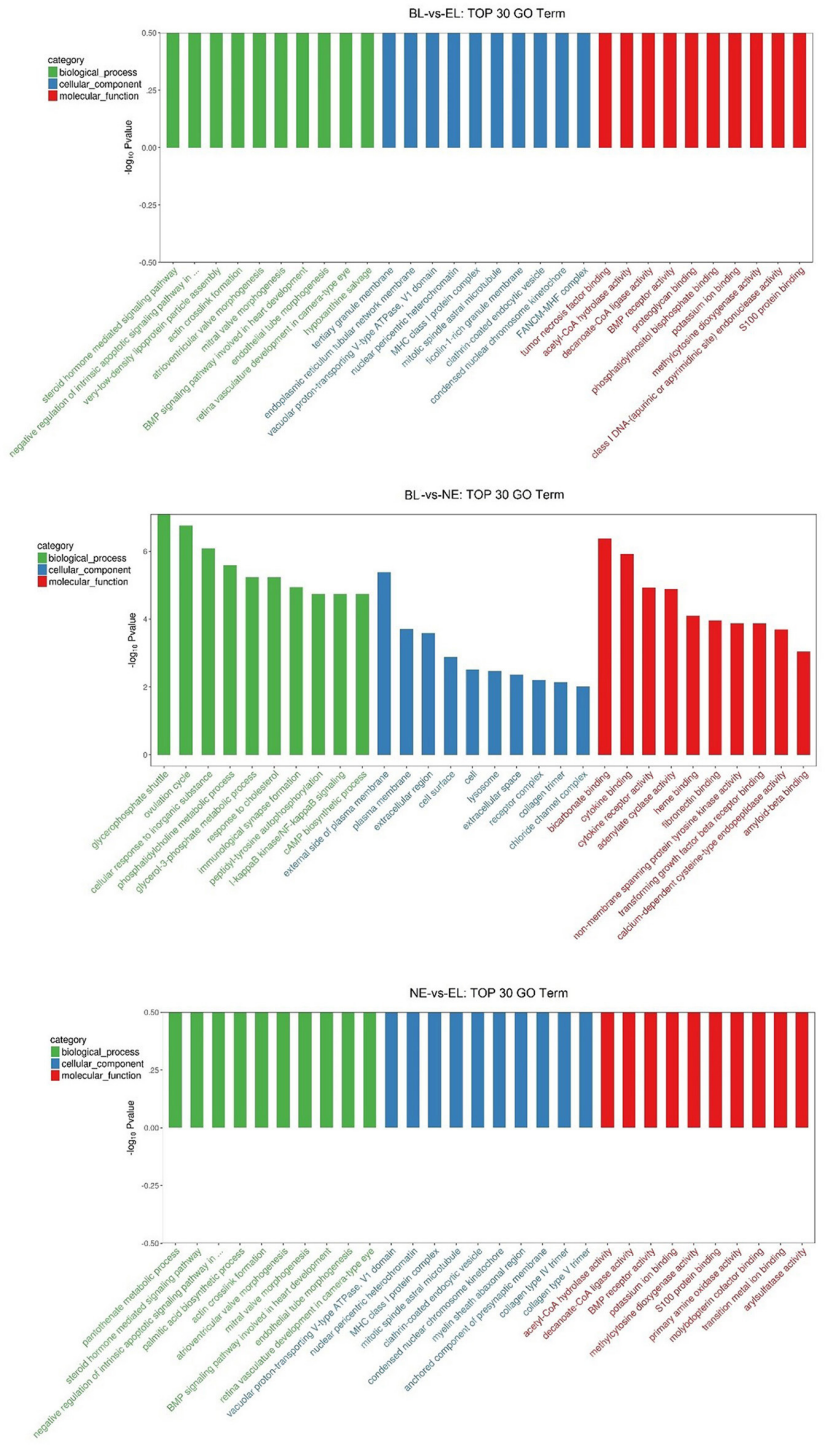
Top 30 GO terms for biological processes, cellular component and molecular function in three comparisons.

Gene Ontology (GO) enrichment analysis was performed to further express the functional roles of DEGs in ovarian development. The top 30 most significant GO terms for biological processes (BP), cellular component (CC), and molecular function (MF) in the pairwise three comparisons are shown in Figure 5. The most significant GO terms related to ovarian development in BL-vs.-EL included “steroid hormone-mediated signaling pathway” and “actin crosslink formation” in BF, “tertiary granule membrane” and “ficolin-1-rich granule membrane” in CC, and “BMP receptor activity” and “S100 protein binding” in MF. The most significant GO terms related to ovarian development in BL -vs.-NE included “ovulation cycle” and “cAMP biosynthetic process” in BF, “plasma membrane” and “extracellular region” in CC, and “transforming growth factor beta receptor binding” and “cytokine binding” in MF. The top significantly enriched GO terms related to ovarian development in NE-vs.-EL included “steroid hormone-mediated signaling pathway” and “actin crosslink formation” in BF, “collagen type IV trimer” and “clathrin-coated endocytic vesicle” in CC, and “BMP receptor activity” and “decanoate-CoA ligase activity” in MF. However, there was no common TOP GO term notably enriched among the three comparisons.

**Figure 6:**
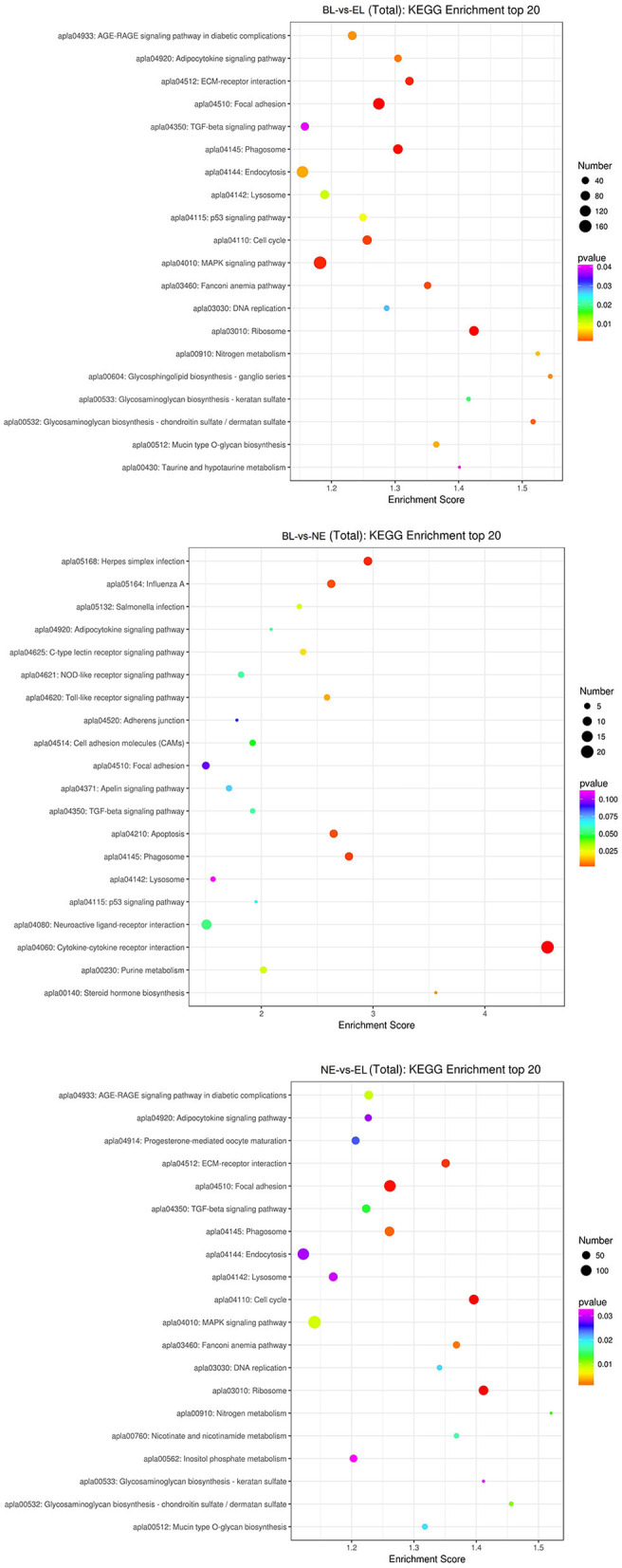
Top 20 KEGG in three comparisons.

**Figure 7:**
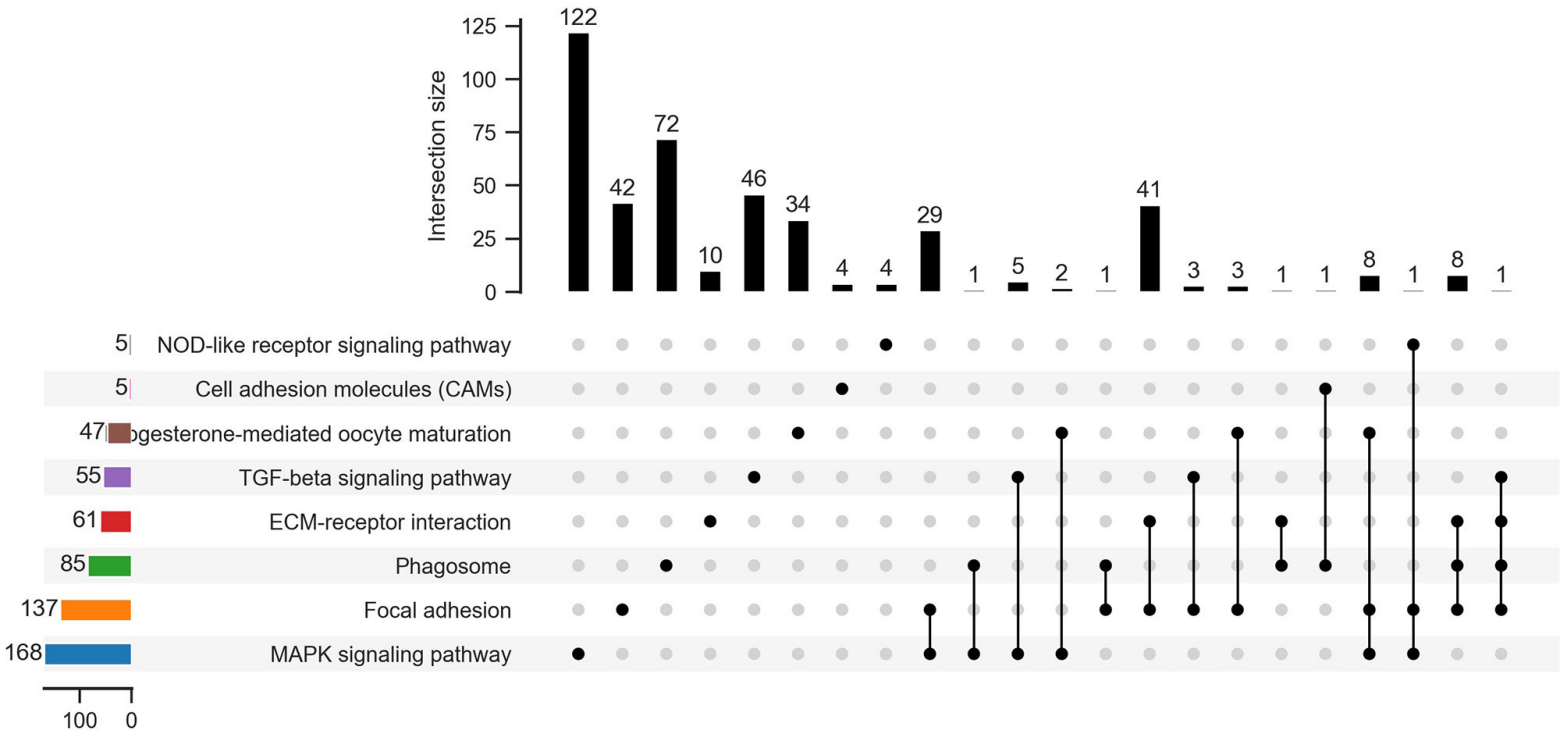
Venn diagram of 439 DEGs enriched among 8 potential pathways.

To better understand the biological functions and interaction of genes, KEGG pathway analysis was performed for the identified DEGs. The top 20 significantly enriched pathways (*P*<0.05) by DEGs of each pairwise comparison are shown in Figure 6, including focal adhesion, MAPK signaling pathway, and TGF-beta signaling pathway in BL-vs.-EL; cytokine–cytokine receptor interaction, TGF-beta signaling pathway, and focal adhesion in BL-vs.-NE; focal adhesion, MAPK signaling pathway, and progesterone-mediated oocyte maturation in NE-vs.-EL. Based on the significance levels of KEGG pathways and the literature reviews of the pathway's function, a total of eight pathways were considered as potential pathways related to egg production and ovarian development of Gaoyou duck, including MAPK signaling pathway, progesterone-mediated oocyte maturation, cell adhesion molecules (CAMs), NOD-like receptor signaling pathway, ECM-receptor interaction, focal adhesion, TGF-beta signaling pathway, and phagosome. All the DEGs enriched in these eight pathways were analyzed through Venn software, and 439 candidate DEGs were screened for further analysis (Figure 7).

### 3.6. Temporal clustering analysis of differentially expressed genes

**Figure 8:**
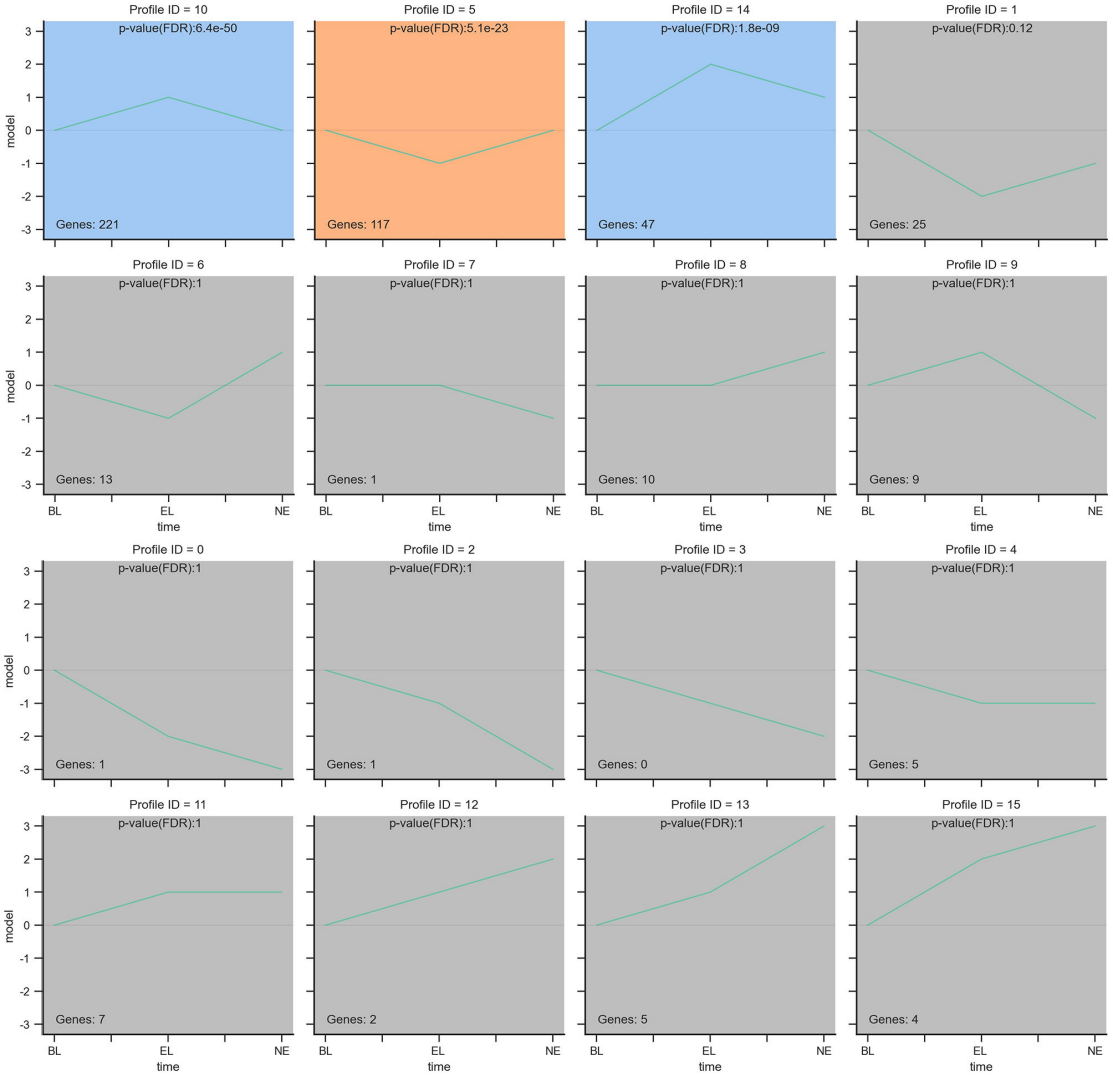
The expression patterns of 468 genes analyzed by model profile. The expression patterns of 468 genes were analyzed and 16 model profiles were used to summarize. Each box represents a model expression profile. 3 expression patterns of genes showed significant *P*-values (*P* < 0.05) (colored boxes).

**Figure 9:**
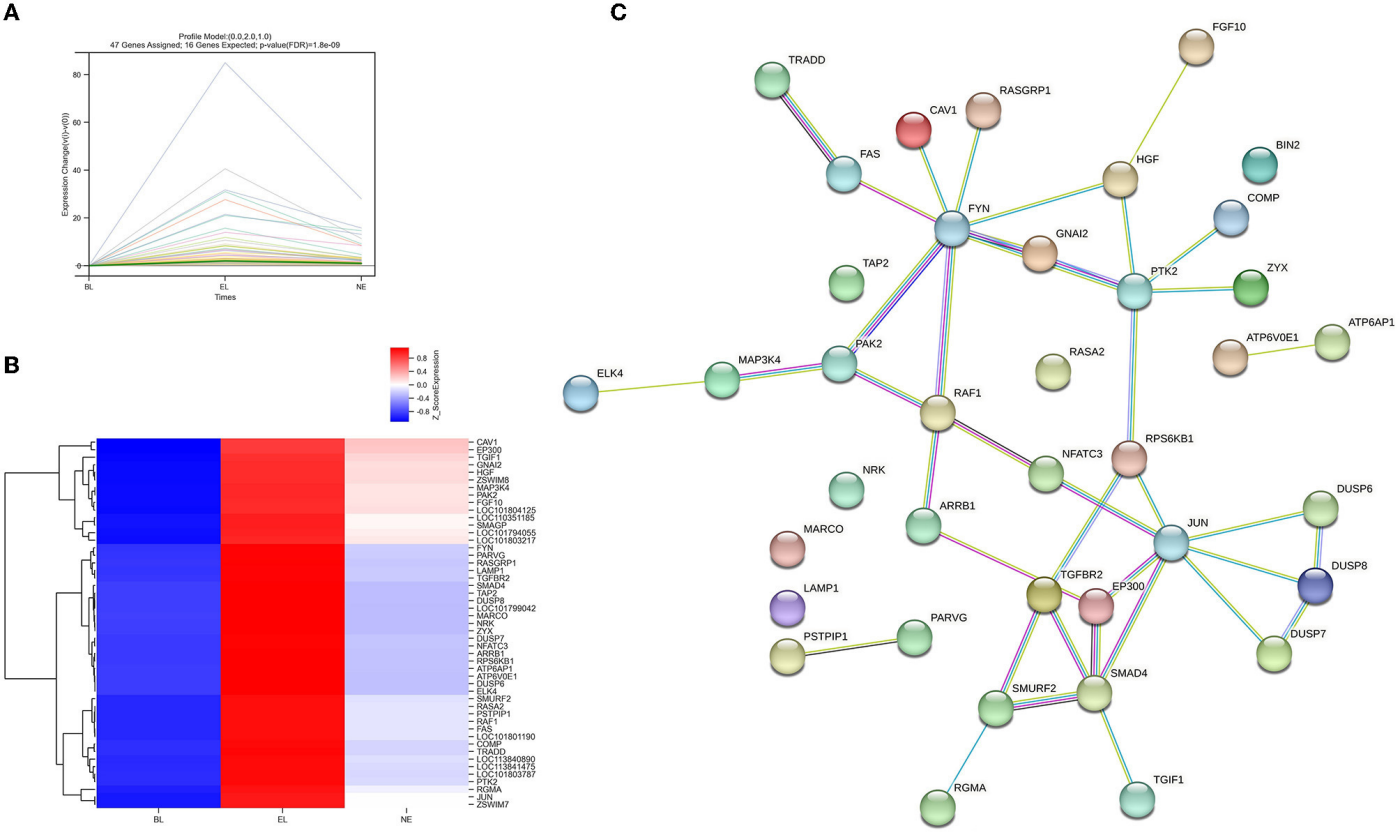
Genes of profile NO.14 of Gaoyou ducks' ovaries at different physiological stages. **(A)** 47 genes expression profile of NO.14, the horizontal axis represents different physiological stages of Gaoyou duck's ovary, and the vertical axis shows the time series of gene expression levels for the gene after normalized transformation. **(B)** Heat map showing the differential gene expression of profile NO.14. “blue” represents low relative expression level, and “red” represents high relative expression level. Each column and row represent a sample, and a gene respectively. **(C)** PPI network of 47 genes based on the STRING database.

Furthermore, considering the significance levels of the top 29 DEGs and the 439 DEGs enriched by KEGG pathway results, a total of 468 candidate DEGs were screened from RNA-Seq results of Gaoyou duck ovaries at three different physiological stages. To further narrow candidate genes which harbor great significance, we summarized and clustered the expression patterns of these 468 genes. As shown in Figure 8, among the 16 patterns, we identified three patterns of genes with significant *p-*values (colored boxes) (numbers 5, 10, and 14), which contained 221, 117, and 47 genes, respectively. Considering ovary development and function at different stages, we focused on profile NO.14. A protein–protein interaction (PPI) network based on the STRING database was generated to visualize the relationship between the 47 potential candidate genes (Figure 9). Based on the PPI network and literature reviews, TGIF1, TGFBR2, RAF1, PTK2, and FGF10 were selected as potential candidate genes for ovarian function and follicular development of Gaoyou duck.

## 4. Discussion

The egg-laying process of poultry is a complex developmental biological process, highly affected by the characteristics of the ovarian tissue. The function of the ovary at different development stages directly affects egg production through various genes, which may have different expressions under different physiological conditions (13). In this study, the ovary transcriptomes, which include age at 150 days before laying (BL), 240 days of egg laying (EL), and 500 days of nesting (NE), were analyzed through RNA-Seq to deepen our understanding of Gaoyou duck laying performance.

Based on the transcriptomic studies, 2,782 genes were differentially expressed between the BL and EL groups; 46 and 2,516 DEGs were discovered during the pairwise comparisons of BL-vs.-NE and NE-vs.-EL, respectively. To validate the reliability of the transcriptomic sequencing results, six DEGs were randomly selected for qRT-PCR verification from the original tissue, and the expression levels proved that the six genes were consistent with the RNA-Seq data. The qRT-PCR verification results indicated that the RNA-Seq data and the screened DEGs were highly reliable and could be used for further analysis.

Pathway functional analysis found that focal adhesion, phagosome, cell adhesion molecules (CAMs), MAPK signaling pathway, and regulation of actin cytoskeleton were involved in the whole reproductive process of Gaoyou duck. Focal adhesion is a special structure formed at the contact site between cells and the extracellular matrix, which is used as a combinatorial site for creating different signaling complexes for cells (14). Focal adhesion participates in cell migration, proliferation, and differentiation. Focal adhesion has been identified in previous ovary transcriptome studies, including Jinghai yellow chicken (13), white Muscovy duck (15), and Xinjiang Yili Geese (16). An increasing body of evidence suggests that the MAPK (mitogen-activated protein kinase) signaling pathway participates in cell proliferation, differentiation of apoptosis, and the reproductive process (17). A recent study identified that the MAPK signaling pathway was involved in the egg production and folliculogenesis of Indian domestic ducks (4). The actin cytoskeleton signaling pathway is involved in cellular functions (18). Through actin cytoskeleton reorganization, the proliferation of granulosa cells could be promoted (19). Cheng et al. (20) treated murine ovaries with an actin polymerization-promoting cyclic peptide and discovered that follicle growth was stimulated both *in vivo* and *in vitro*. In duck's (6), goose's (21), and goat's (22) ovary transcriptome studies, the actin cytoskeleton has been discovered to be related to ovary function. The phagosome and cell adhesion molecules (CAMs) were consistently reported in a study comparing the ovary of high- and low-laying ducks (23). Overall, these signaling pathways may control ovary development and may be involved in the egg production of Gaoyou duck.

Transforming growth interaction factor 1 (TGIF1) is one of the TALE homolog domain protein family members which participate in many physiological processes including lipid and carbohydrate metabolism (11), inflammatory reaction (24, 25), and inhibition of androgen receptor activity (26). An et al. (27) revealed an insight that TGIF1 could suppress the secretion of E2/P4 in goat's granulosa cells and inhibited the apoptosis of granulosa cells. Martin (28) described that TGIF1 is expressed in chicken cortical and pre-granulosa cells and is required for ovarian development. In this study, we provided preliminary evidence that TGIF1 was deferentially expressed during the development of the Gaoyou duck ovary, suggesting that it may also play a role in ovarian function and follicular development.

Transforming growth factor beta receptor II (TGFBR2) represents a core member of the TGF-beta signaling pathway that plays an important role in ovary function (29). In 1993, Tsuchina et al. (30) found that TGFBR2 mRNA is expressed in the porcine granulosa cells of rat ovaries. Moreover, Roy (31) discovered that TGFBR2 is expressed in human ovarian granulosa cells, corpus luteum cells, and interstitial cells. A study by Li et al. (32) showed that TGFBR2 is highly expressed in the Erhualian pig's ovary. Li (33) compared high- and low-fecundity groups of Hu sheep and found that the expression of EGFBR2 is higher in the ovary than in other tissues and is significantly higher in the high-fecundity group than in the low-fecundity group. Through TGFRB2^gc−/−^ mice, Yang et al. (34) found that TGFRB2 deletion could impair oocyte meiotic arrest, ovulation, and female fertility. In the present study, we found that ovarian TGFBR2 expression was higher during the egg-laying period than the other two periods, suggesting that it might be involved in the egg-laying performance of the Gaoyou duck.

V-RAF-leukemia viral oncogene 1 (RAF1) is the core member of the RAS/RAF/MEK/ERK signaling pathway and has been approved to play an essential role in reproduction and development by regulating steroid hormone synthesis (35, 36). Through *in vitro* and *in vivo* studies, Luo et al. (37) found that RAF1 could stimulate E2 synthesis and secretion through the FSH signaling pathway in mouse granulosa cells. Nonetheless, the role of RAF1 in ovarian function and follicular development of poultry has been largely understudied. Based on its function in regulating steroid hormone synthesis, it can be affirmed that RAF1 is essential in Gaoyou duck ovary development.

Protein tyrosine kinase 2 (PTK2) is a member of the PTK family which plays an important role in cell–cell contact by junction formation between cells (38–40). PTK2 has been extensively linked with tumor progression, especially in ovarian cancer (41, 42). Shabbir et al. (43) compared genome-wide transcriptome profiling of 1 month, 3 months, and 8 months Hu sheep's ovaries, in which PTK2 was identified as the target gene of screened essential LncRNAs involved in ovarian and follicular development. By developing an oocyte-specific PTK2-knockout mouse model, PTK2 was indispensable in the communication of oocyte and granulosa cells, which has huge implications for male and female gametogenesis. Due to the importance of PTK2 in gametogenesis, it can be inferred that this gene could participate in the ovarian and follicular development of ducks.

Fibroblast Growth Factor 10 (FGF10) is one of the most important members of the Fibroblast Growth Factor family, which participates in many cellular processes (44). The transcription level of FGF10 has been studied in bovine (45), goat (46), and human ovaries (47), based on which FGF10 is one of the regulators of steroidogenesis and follicular development of the ovary. Oron et al. (48) discovered that FGF10 could be involved in the initial follicular development in humans. Moreover, Chaves et al. (46) cultured goat ovaries with FGF10 *in vitro* and confirmed that FGF10 could stimulate the growth of follicles. Similarly, Du et al. (49) cultured buffalo oocytes with FGF10 *in vitro* and found that an appropriate concentration of FGF10 was conducive to oocyte maturation and embryo development. In this study, FGF10 is highly expressed in the egg-laying period, substantiating the importance of FGF10 in the egg production of Gaoyou ducks.

## 5. Conclusion

Ovary development is a dynamic process that affects follicle formation and egg-laying performance. This study identified eight important pathways and five candidate genes contributing to ovarian development in Gaoyou ducks. These findings lay the foundation for a better understanding of egg production, especially double-yolk egg production in Gaoyou ducks.

## Data availability statement

The data presented in the study are deposited in the Sequence Read Archive in NCBI, accession number PRJNA940302.

## Ethics statement

The animal study was reviewed and approved by Experimental Animal Ethics Committee of Jiangsu Agri-animal Husbandry Vocational College (Ethic approval file No. JAHV-2020-58).

## Author contributions

JW: conceptualization. GS and LZ: methodology. GS and JX: software. LZ and XL: validation. LZ: formal analysis, data curation, writing—original draft preparation, supervision, and project administration. LZ and RJ: investigation. LZ and JW: resources and funding acquisition. LZ and JX: writing—reviewing and editing. LZ and XZ: visualization. All authors have read and agreed to the published version of the manuscript.
