# Supplementary material for: Identification of differentially expressed genes and signaling pathways in Gaoyou duck ovary at different physiological stages
Source: Front Vet Sci. 2023 May 3;10:1190998. doi: 10.3389/fvets.2023.1190998 (PMC10189055; doi:10.3389/fvets.2023.1190998)
Supplement: Supplementary file 1 [file Data_Sheet_1.docx]

Supplementary Material

Identification of differentially expressed genes and signalling pathways in Gaoyou duck ovary at different physiological stages

Lei Zhang ^a1^, Jun Xie ^a1^, Guobo Sun ^a^, Rongchao Ji ^a^, Xiaoming Li ^a^, Xue Zhang ^a^ and Jian Wang ^a^*

^a^Jiangsu Agri-animal Husbandry Vocational College, Taizhou 225300, China.

^1^These authors contributed equally to this work.

*** Correspondence:**Corresponding Author
[568418583@qq.com](mailto:568418583@qq.com;)

# Supplementary Tables

**Table S1 Primer information for qRT-PCR**

| Gene | Sequence (5′-3′) | Tm (°C) |
| --- | --- | --- |
| *KIT* | F: AGAAGCCACTGCTTACGGTC | 58 |
|  | R: ACCAGTGTGGGTCCTCCAAT |  |
| *IGF1* | F: TGACATTGCTCTCAACATCTCAC | 60 |
|  | R: GGTAAGCAAACACAGGCCAAG |  |
| *COL3A1* | F: CCGTGCCTCCCAGAACATTA | 56 |
|  | R: ACCACAGGCAACCTCATTGT |  |
| *LOC101793561* | F: CAGGCTGCCAAGAATGGAGA | 58 |
|  | R: ACGGTGAGTGAAATGCACCT |  |
| *DLGAP3* | F: CCGACGACAATTTGGACAGC | 60 |
|  | R: CCTTCCCCACCGCTCTTTAG |  |
| *LOC101793529* | F: GTCCTGGCCGTAGTCATGTT | 60 |
|  | R: TGAGAAAGGTGTTGCCCAGT |  |
| *GADPH* | F：TCGGAGTCAACGGATTTGGC | 60 |
|  | R：TTCTCAGCCTTGACAGTGCC |  |

**Table S2 Filtered data for RNA-Seq**

| Sample | raw_reads（Mb） | raw_bases (Gb) | clean_reads (Mb) | clean_bases (Gb) | valid_bases (%) | Q30 (%) | GC (%) |
| --- | --- | --- | --- | --- | --- | --- | --- |
| BL1 | 56.24 | 8.44 | 54.71 | 7.88 | 93.42 | 94.73 | 47.09 |
| BL2 | 55.61 | 8.34 | 54.30 | 7.83 | 93.83 | 95.27 | 47.38 |
| BL3 | 55.74 | 8.36 | 54.41 | 7.86 | 93.95 | 95.21 | 47.32 |
| EL1 | 51.96 | 7.79 | 50.61 | 7.23 | 92.79 | 94.65 | 49.27 |
| EL2 | 51.92 | 7.79 | 50.46 | 7.24 | 92.97 | 94.47 | 49.10 |
| EL3 | 50.86 | 7.63 | 49.53 | 7.11 | 93.20 | 94.70 | 49.06 |
| NE1 | 57.65 | 8.65 | 56.31 | 8.09 | 93.51 | 95.34 | 48.33 |
| NE2 | 59.28 | 8.89 | 57.88 | 8.40 | 94.42 | 95.35 | 47.05 |
| NE3 | 53.67 | 8.05 | 52.52 | 7.48 | 92.94 | 95.51 | 47.93 |

Q30 represents the percentage of bases with Phred value more than 30 in the total bases, indicating that the correct recognition rate of bases is 99.9%; GC represents the percentage of the total number of G and C in clean bases.
